# Bioactivity-Guided Fractionation of an Antidiarrheal Chinese Herb *Rhodiola kirilowii* (Regel) Maxim Reveals (-)–Epicatechin-3-Gallate and (-)–Epigallocatechin-3-Gallate as Inhibitors of Cystic Fibrosis Transmembrane Conductance Regulator

**DOI:** 10.1371/journal.pone.0119122

**Published:** 2015-03-06

**Authors:** Lei Chen, Bo Yu, Yaofang Zhang, Xin Gao, Liang Zhu, Tonghui Ma, Hong Yang

**Affiliations:** 1 School of Life Sciences, Liaoning Provincial Key Laboratory of Biotechnology and Drug Discovery, Liaoning Normal University, Dalian, 116029, P. R. China; 2 College of Basic Medical Sciences, Dalian Medical University, Dalian, 116044, P. R. China; 3 School of Medicine, Yanbian University, Yanji, 133002, P. R. China; Jilin University, CHINA

## Abstract

Cystic fibrosis transmembrane conductance regulator (CFTR) is the principal apical route for transepithelial fluid transport induced by enterotoxin. Inhibition of CFTR has been confirmed as a pharmaceutical approach for the treatment of secretory diarrhea. Many traditional Chinese herbal medicines, like *Rhodiola kirilowii* (Regel) Maxim, have long been used for the treatment of secretory diarrhea. However, the active ingredients responsible for their therapeutic effectiveness remain unknown. The purpose of this study is to identify CFTR inhibitors from *Rhodiola kirilowii* (Regel) Maxim via bioactivity-directed isolation strategy. We first identified fractions of *Rhodiola kirilowii* (Regel) Maxim that inhibited CFTR Cl^-^ channel activity. Further bioactivity-directed fractionation led to the identification of (-)–epigallocatechin-3-gallate (EGCG) as CFTR Cl^-^ channel inhibitor. Analysis of 5 commercially available EGCG analogs including (+)–catechins (C), (-)–epicatechin (EC), (-)–epigallocatechin (EGC), (-)–epicatechin-3-gallate (ECG) and EGCG revealed that ECG also had CFTR inhibitory activity. EGCG dose-dependently and reversibly inhibited CFTR Cl^-^ channel activity in transfected FRT cells with an IC_50_ value around 100 μM. In *ex vivo* studies, EGCG and ECG inhibited CFTR-mediated short-circuit currents in isolated rat colonic mucosa in a dose-dependent manner. In an intestinal closed-loop model in mice, intraluminal application of EGCG (10 μg) and ECG (10 μg) significantly reduced cholera toxin-induced intestinal fluid secretion. CFTR Cl^-^ channel is a molecular target of natural compounds EGCG and ECG. CFTR inhibition may account, at least in part, for the antidiarrheal activity of *Rhodiola kirilowii* (Regel) Maxim. EGCG and ECG could be new lead compounds for development of CFTR-related diseases such as secretory diarrhea.

## Introduction

Maintenance of an appropriate amount of intestinal fluid is vital for digestion and clearance of the luminal contents. It is a passive process driven by the active anion, predominantly Cl^-^, transport from blood to the intestinal lumen [1, 2]. The major components in fluid secretion involve Cl^-^ intake via Na^+^/K^+^/2Cl^-^ cotransporter (NKCC1) through the basolateral membrane and Cl^-^ exit to the lumen via cystic fibrosis transmembrane conductance regulator (CFTR) and Ca^2+^-activated Cl^-^ channels (CaCCs) in apical membrane of secretory epithelial cells [1, 3, 4].

CFTR belongs to the superfamily of ATP-binding cassette (ABC) proteins, whose core units contain two membrane-spanning domains (MSDs) and two nucleotide-binding domains (NBDs). CFTR contains a regulatory (R) region, which is unique to this superfamily. Activity of CFTR is regulated by binding and hydrolysis of ATP at NBDs and by phosphorylation of the R region [5, 6]. Though CFTR is not the sole pathway for apical Cl^-^ exit, it is the predominant pathway for Cl^-^ transport in active fluid secretion evoked by cholera toxin and *E*. *coli* heat-stable enterotoxin [7–9]. CFTR is a well-validated target for development of inhibitors for therapy of secretory diarrheas [10–12].

Small-molecule blockers of CFTR have been proven valuable for the development of drugs to treat cholera and traveler’s diarrhea [13, 14]. So far, several CFTR inhibitors have been identified and characterized [10, 15–19], among which the most prominent one is the thiazolidinone CFTR_inh_-172, a CFTR selective blocker identified from a combinatorial small molecule library. Though CFTR_inh_-172 is highly specific to CFTR protein and could potently reduce cholera toxin-induced intestinal fluid secretion in rodents, poor water solubility (<5 μM) of the compound greatly limits its potential use in the treatment of diarrhea [20].

Natural products have long been the major resources for new drugs, and many successful drugs originated from natural compounds [21–23]. Natural compounds are highly diverse in structure and often provide highly specific biological activities [24–26]. Traditional Chinese herbal medicine contains large numbers of therapeutic compounds for a broad spectrum of human diseases including secretory diarrhea. Systematic investigation on the pharmacology of active ingredients and mechanisms are crucial for transforming traditional herbal practices into evidence-based medicine.

We report here the identification of CFTR Cl^-^ channel inhibitors from a traditional Chinese herbal antidiarrheal medicine. We found two galloyl-containing catechins (EGCG and ECG) as CFTR inhibitors. Galloyl-containing catechins are major components of *Rhodiola kirilowii* (Regel) Maxim and green tea that have been reported to have many biological (mainly anticancer and cancer-preventive) activities. Here, we report a new activity for EGCG and ECG, providing a molecular mechanism for the antidiarrheal efficacy of *Rhodiola kirilowii* (Regel) Maxim.

## Results

### CFTR inhibition by fractions of *Rhodiola kirilowii* (Regel) Maxim

**Fig 1:**
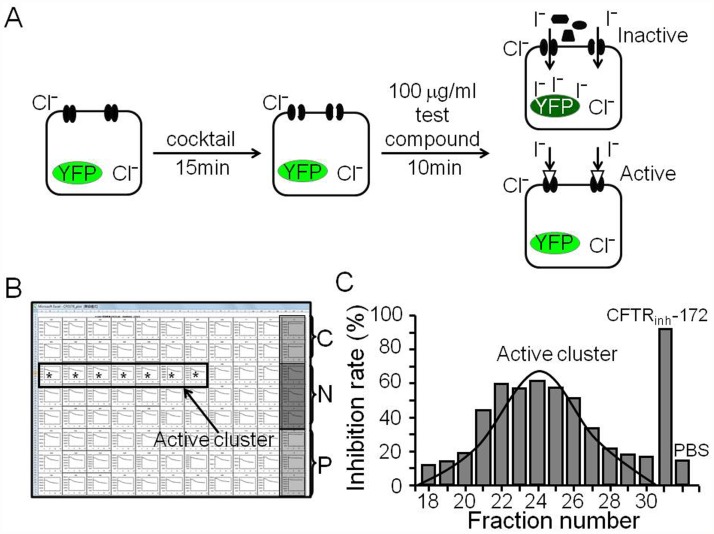
Inhibition of CFTR Cl^¯^ channels by fractions from *Rhodiola kirilowii* (Regel) Maxim. A. Principle of cell-based fluorescence assay. FRT cells stably co-transfected with human CFTR and YFP-H148Q were prestimulated with a cocktail (5 μM FSK, 100 μM IBMX and 25 μM GEN) for 10 min before addition of a test sample to final concentration of 100 μg/ml. CFTR-mediated I^¯^ influx was measured from the kinetics of decreasing YFP-H148Q fluorescence in response to addition of I^¯^ solution. B. Time-course fluorescence data from a 96-well microplate of CFTR-expressing FRT cells. Controls (C: PBS; N: cocktail; P: cocktail plus 20 μM CFTR_inh_-172) are shown on the right. Asterisk show wells with decreased I^¯^ influx (representing CFTR inhibition). C. % inhibition rate of the active wells indicated Gaussian distributions of the active sites.

*Rhodiola kirilowii* (Regel) Maxim was extracted using 95% ethanol on Soxhlet reflux apparatus, and then the extract was fractionated into 80 fractions by preparative HPLC with a linear gradient of 0–90% methanol (MeOH). The fractions were dried and dissolved in DMSO to generate 5 mg/ml solutions in a 96-well plate. To identify CFTR inhibitors, we used a cell-based fluorescence assay (Fig. 1A). The analysis revealed inhibition of CFTR-mediated I^-^ influx by a cluster of fractions (Fig. 1B). Fig. 1C shows that fractions 21–27 significantly inhibited CFTR-mediated I^-^ influx; whereas the other fractions had little effect.

### Bioactivity-directed fractionation and structure determination

**Fig 2:**
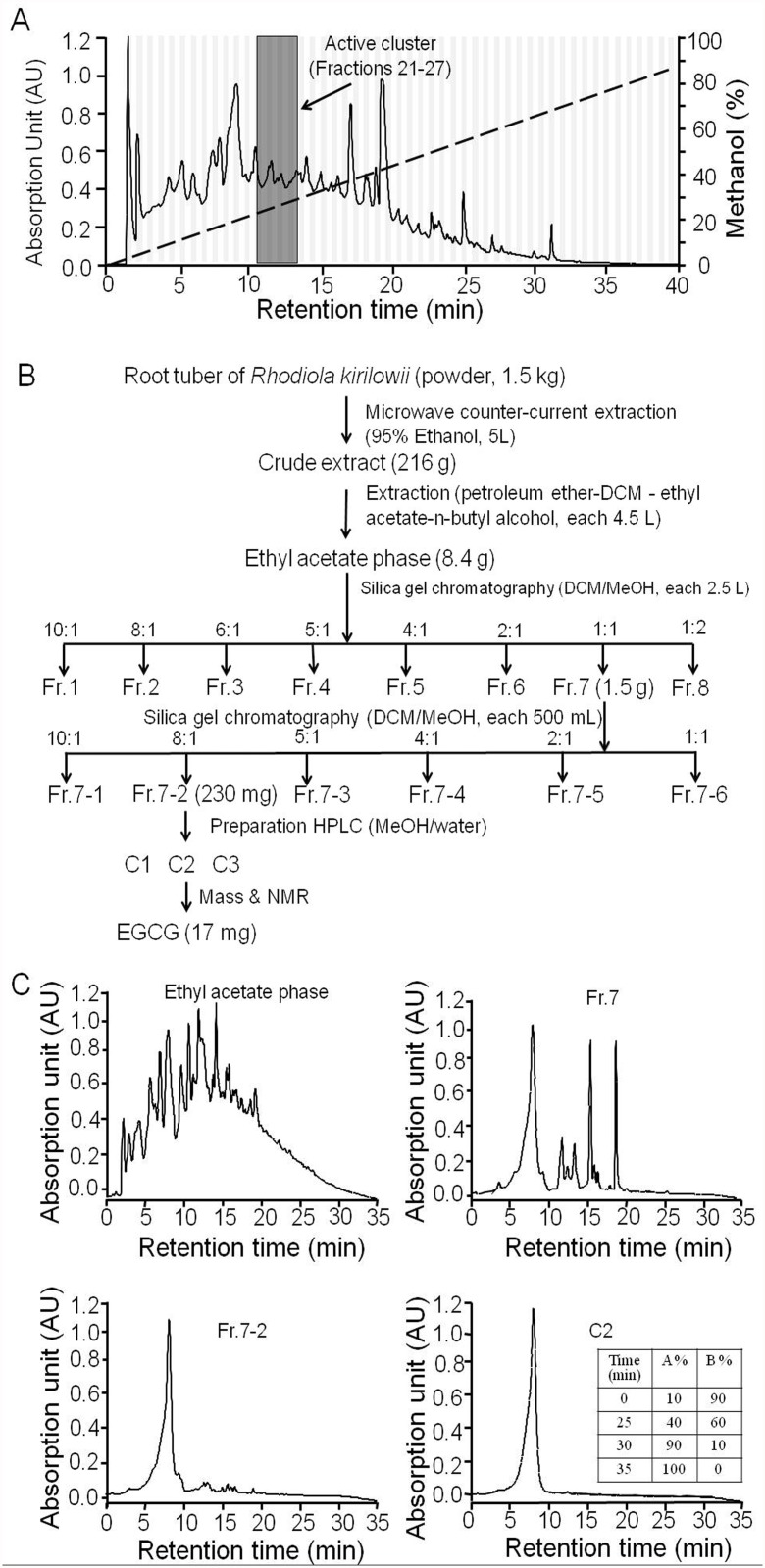
Fractionation of *Rhodiola kirilowii* (Regel) Maxim. A. HPLC fractionation of *Rhodiola kirilowii* (Regel) Maxim showing the chromatogram at 220 nm absorbance. Vertical bars show 80 collected fractions. B. Activity-directed fractionation scheme for the isolation CFTR inhibition compounds from *Rhodiola kirilowii* (Regel) Maxim. C. Analytical HPLC chromatograms of fraction ethyl acetate phase, Fr.7, Fr.7–2, and C2 at 220 nm absorbance. The gradient of analytical HPLC was developed with mobile phase A (acetonitrile) and mobile phase B (0.2% acetic acid) at 0.2 mL/min flow rate (inset)

Fig. 2A shows preparative HPLC chromatogram of the *Rhodiola kirilowii* (Regel) Maxim 95% ethanol extract. The active fractions 21–27 (appeared as a clusters) were eluted at 11–14 min. We next optimized extraction and purification methods for CFTR inhibitor isolation under bioactivity-directed strategy. *Rhodiola kirilowii* (Regel) Maxim was first extracted with 95% ethanol by using microwave extraction equipment, and then the crude extract was extracted sequentially using a series of solvents including petroleum ether, dichloromethane (DCM), ethyl acetate, and n-butyl alcohol. The plate reader assay indicated that the ethyl acetate fraction was the most effective one, so, the fraction was subjected to further isolation. After two cycles of silica gel chromatography, two active fractions (Fr.7 and Fr.7–2) were obtained, respectively. The active fraction Fr.7–2 was further purified by HPLC to get active fraction C2. Detailed activity-directed fractionation protocol was seen in the Material and Method section and summarized in Fig. 2B. Analytical HPLC chromatograms of ethyl acetate extract, Fr.7, Fr.7–2, and C2 were shown in Fig. 2C.

**Fig 3:**
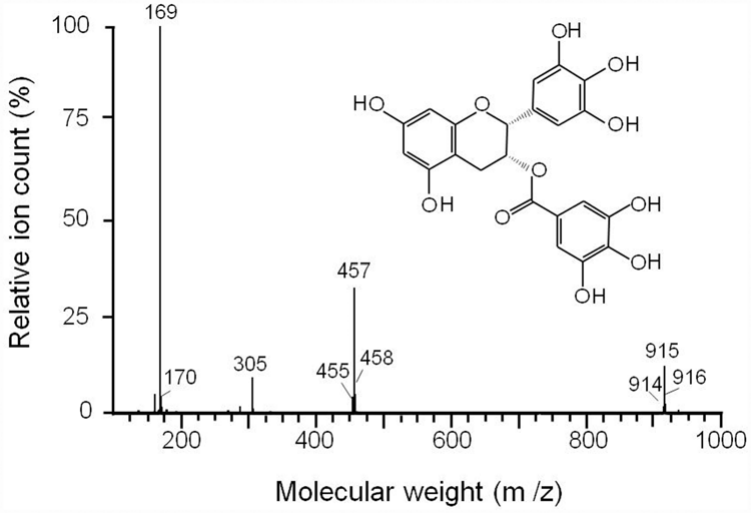
Identification of purified active fraction as EGCG. Mass spectra of purified fraction C2. Inset: Structure of EGCG.

Because fraction C2 shows a single peak with a purity >99%, the compound was applied to mass spectrometry (MS) analysis, which gave the molecular size (458.4 Da) and known fragmentation pattern of EGCG (Fig. 3). Nuclear magnetic resonance (NMR) spectrometry gave the ^1^H and 13C pattern identical to EGCG, which further confirmed the molecule identity of the active compounds as EGCG.

### Structure-activity analysis

**Fig 4:**
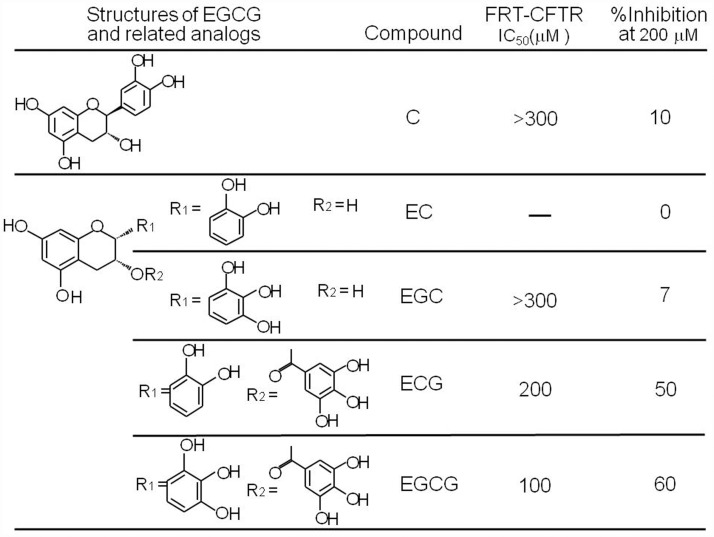
Structure-activity analysis of catechin analogs. IC_50_ was determined from concentration-inhibition data from cell-based fluorescence measurements.

Next, we analyzed the effects of commercially available EGCG and 4 structural analogs (C, EC, EGC, and ECG) on CFTR Cl^-^ channel activities by fluorescent quenching assay. The results confirmed the activity of EGCG and found that ECG also exhibited significant CFTR inhibitory activity. Fig. 4 summarized the structures and activities of EGCG and 4 analogs. Further time-course results indicated that rapid inhibition of CFTR Cl^-^ channel activities by EGCG and ECG, with half-maximal activation in 5 min; and the inhibitions could be fully reversed at ~30 min after EGCG and ECG washout (data not shown).

### Biological studies

**Fig 5:**
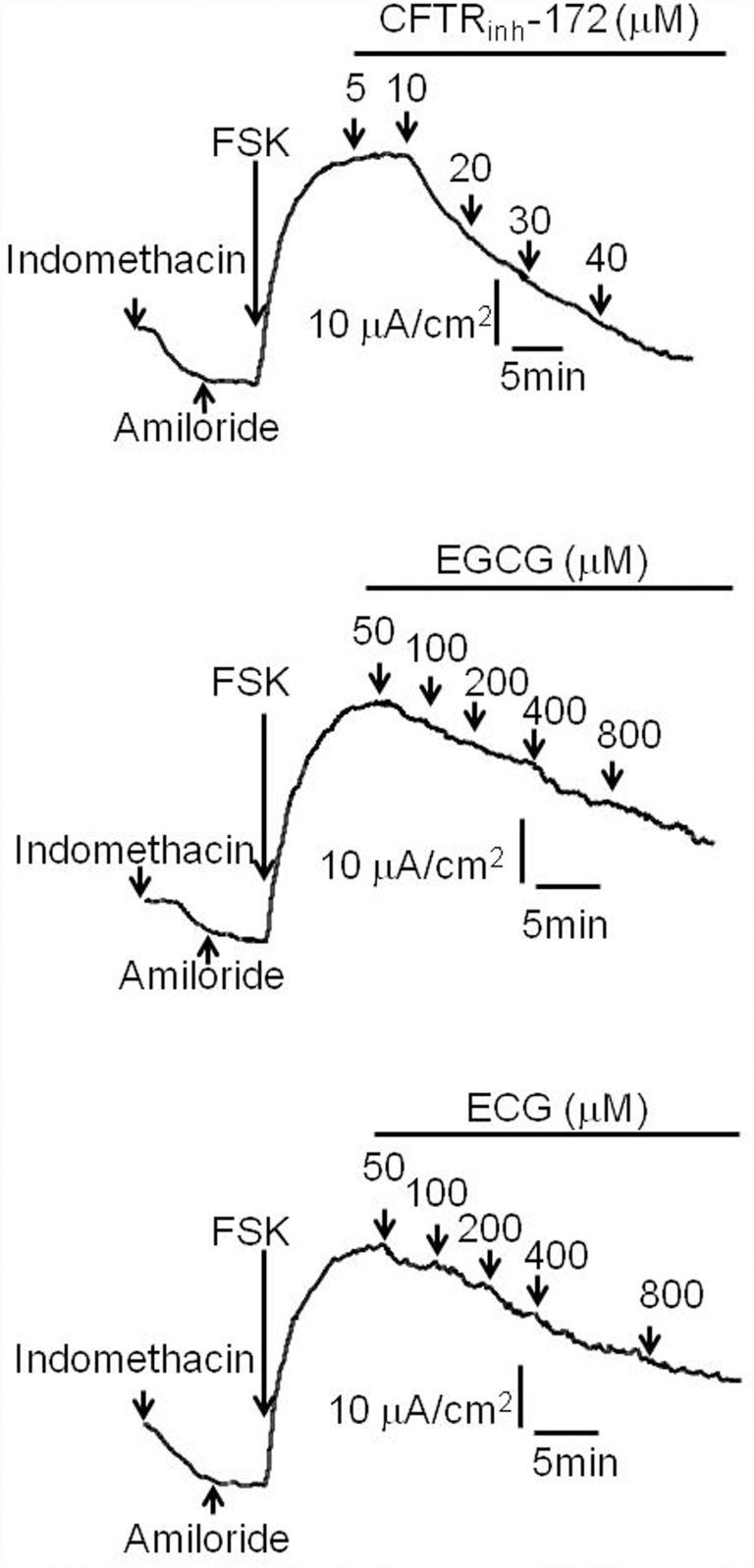
CFTR inhibition by EGCG and ECG. **A.** Dose—response relationship of EGCG and ECG determined in the iodide influx assay. Data were expressed as mean±SE, n = 3). B. EGCG and ECG inhibition short-circuit current after amiloride and indomethacin addition and stimulation by FSK (20 μM) in isolated rat colonic mucosa. EGCG and ECG were added to mucosal surfaces as indicated. One experiment typical of four or five is shown.

CFTR is expressed in the crypt cells in the distal small intestine and colon, and it is the major pathway for Cl^-^ secretion into intestinal lumen [4, 27]. The efficacies of EGCG and ECG were tested *ex vivo* in isolated rat colonic mucosa by Ussing chamber short-circuit assay with CFTR_inh_-172 as a positive control. Amiloride (10 μM) and indomethacin (10 μM) were present in the chamber solutions for entire experimental periods to prevent Na^+^ current and prostaglandin generation. As shown in Fig. 5, EGCG and ECG dose-dependently inhibited short-circuit currents in intact rat colonic mucosa.

**Fig 6:**
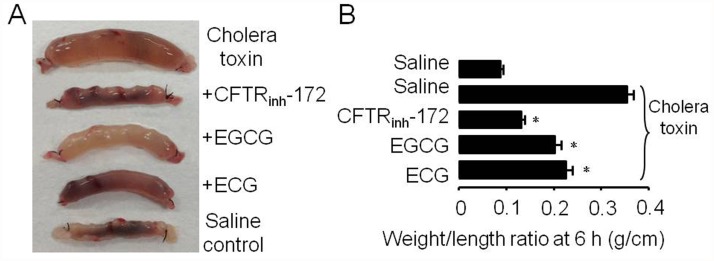
Inhibition of intestinal fluid secretion by EGCG and ECG. **A.** Photograph of isolated mouse ileal loops at 6 hours after lumenal injection of saline, 0.5 μg cholera toxin, 0.5 μg cholera toxin plus 10 μg EGCG (or 10 μg ECG, or 2 μg CFTR_inh_-172). (B) Ratio of loop weight/length (g/cm) at 6 hours before versus after luminal fluid removal (SE; six mice per group;*p < 0.001).

Next, we investigated the *in vivo* efficacies of EGCG and ECG in inhibiting cholera toxin—induced intestinal fluid secretion in live mice. As shown in the images in Fig. 6A and summarized data in Fig 6B. Massive fluid was accumulated in cholera toxin—treated loops as compared to the saline control group. Intraluminal injection of EGCG (10 μg) and ECG (10 μg) significantly reduced cholera toxin—induced intestinal fluid secretion as quantified from loop weight-to-length ratio, supporting that EGCG and ECG are active antidiarrheal ingredients of *Rhodiola kirilowii* (Regel) Maxim through inhibition of CFTR chloride channel.

## Discussion

The system of Chinese herbal medicine is built on the experience of herb users over thousands of years. The efficacies of the herbs in many diseases are already well documented [28, 29]. However, because of insufficient studies on material basis and the pharmacology of traditional Chinese medicine (TCM), the molecular mechanisms responsible for their therapeutic effectiveness are still largely unclear. Before TCM has been accepted globally as complementary and alternative medicine for disease treatment and prevention, it is crucial to understand the molecular basis for their effects [30]. To understand the therapeutic mechanisms of TCM, it is critical to identify the active compounds against molecular targets that participate in a particular disease.

The purpose of this study is to identify CFTR inhibitors from traditional Chinese herbal antidiarrheal medicine. Two natural small molecules, EGCG and ECG, were identified as CFTR inhibitors. EGCG and ECG inhibited CFTR Cl^-^ currents in CFTR-transfected FRT cells and isolated rat colonic mucosa. *In vivo*, both compounds effectively reduced cholera toxin-induced intestinal fluid secretion in mouse closed-loop model. Their inhibitory effects on CFTR Cl^-^ currents and intestinal fluid secretion may provide a molecular basis for the antidiarrheal properties of *Rhodiola kirilowii* (Regel) Maxim [31, 32].

One of the fundamental advantages in TCM is the multicomponent therapy, in which several active compounds interact with one molecular target and/or one active compound interacts with several different molecular targets simultaneously. EGCG and ECG, belonging to galloyl-containing catechins, are found in a wide range of natural products and extracts. These compounds have been reported to have various biological activities, including reduced incidence of cardiovascular disease, diabetes, cancer, stroke, cataracts, inflammation, and obesity [33, 34]. Previous studies have shown that catechins had potent antibacterial effects against a number of bacterial pathogens including *Vibrio cholerae* and *Escherichia coli* [32, 35]. Here, we found that EGCG and ECG significantly reduced cholera toxin—induced fluid secretion, suggesting that these compounds may protect patients against *Vibrio cholerae* infection disease via multiple pathways. The major pathways for Cl^-^ transports in intestine include CFTR and CaCC [4, 27, 36]. Namkung et al [36] reported that EGCG and ECG inhibited TMEM16A/CaCC channel activity. EGCG and ECG had inhibitory effect on both CFTR and CaCC Cl^-^ channel activities suggested that mild inhibition of CFTR and CaCC may result in a significant inhibition of intestinal salt and water secretion. These results further support the proposition that TCM are multicomponent and multi-target agents. Elucidation of multicomponent activity of TCM is critical not only for developing new therapeutic strategies to conquer complex diseases, but also for reducing side effects of drugs. EGCG is more soluble in water compared to CFTR_inh_-172, with a maximum solubility ~23.6 mg/mL [37]. Recent study indicated that cocrystallization technique could significantly improve the bioavailability of EGCG which may greatly increase the chance to develop EGCG as a therapeutic agent [37].

Core structure of the five tested catechins contains three types of functional groups, hydroxyl, pyrogallol and gallic groups, on a phenyl benzopyrane ring. Structure-activity relationship analysis of the catechins suggested that gallic group on the phenyl benzopyrane ring is needed for CFTR inhibition, as evidenced by the lack of activity of C, EC and EGC. Further studies of medicinal chemistry are required to optimize the CFTR-inhibitory activity of catechin compounds.

In summary, we have identified EGCG and ECG from antidiarrheal herb *Rhodiola kirilowii* (Regel) Maxim as inhibitors of CFTR Cl^-^ channel. As a new molecular target of natural compounds EGCG and ECG, CFTR inhibition may account, at least in part, for the antidiarrheal activity of *Rhodiola kirilowii* (Regel) Maxim. EGCG and ECG could be new lead compounds for developing therapeutics of CFTR-related diseases such as secretory diarrhea and polycystic kidney disease.

## Material and Methods

### Ethics statement

All animal usage procedures in this study were carried out in strict accordance with the recommendations in the Guide for the Care and Use of Laboratory Animals of the National Institutes of Health and were approved by the Liaoning Normal University Committee on Animal Research. All surgery was performed under sodium pentobarbital anesthesia, and all possible efforts were made to minimize suffering.

### Cell lines, animals and compounds

Fischer rat thyroid (FRT) epithelial cells stably expressing human wild-type CFTR and the EYFP (YFP-H148Q) fluorescence indicator proteins were prepared as described in the references [38, 39]. The cells were cultured in F-12 Coon’s modified medium (Sigma Chemical Co. St. Louis, MO. U.S.A.) supplemented with 10% fetal bovine serum, 2 mM L-glutamine, 100 units/ml penicillin, and 100 μg/ml streptomycin, at 37°C.

Forskolin (FSK), iso-butyl-methylxanthine (IBMX), genistein (GEN), amiloride, indomethacin and cholera toxin were purchased from Sigma Chemical Co. (St. Louis, MO). HPLC grade solvents were purchased from Honeywell (NewJersey, USA). Standard compounds [(+)–catechins (C), (-)–epicatechin (EC), (-)–epigallocatechin (EGC), (-)–epicatechin-3-gallate (ECG) and (-)–epigallocatechin-3-gallate (EGCG)] were purchased from Shanghai Tauto Biotech Co., LTD (Shanghai, China). CFTR_inh_-172 was synthesized as described in the reference [40].

Male Kunming (KM) mice and Wistar rats were fed a standard chow diet and housed under specific pathogen-free conditions at Dalian Medical University (Permit Number: SCXK Liao 2008–0002 for mice and SCXK Liao 2013–0003 for rats).

### General HPLC experimental procedures

The HPLC fractionation was performed on a Waters autopurification system equipped with a constant flow pump (Waters 2525), a compensation pump (Waters 515), a dual channel detector (Waters 2487), an automatic collector (Waters 2767), using a C18 reversed-phase column (XTerra Pre, 19*150 mm, 5 mm particle size, USA). The purity of active fractions was analyzed on a Waters Alliance HPLC system (Waters 2695) with a diode assay detector (Waters 2996), using C18 column reversed-phase column (XTerra OBD, 2.1*150 mm, 5 mm particle size, USA).

### Extraction and isolation

The air-dried roots of *Rhodiola kirilowii* (Regel) Maxim (1.5 kg) were grounded and extracted with 95% aqueous ethanol (10 L) by using a microwave extraction equipment (HWC-50L Microwave Extraction Equipment, Tianshui Huayuan Pharmaceutical Equipment Technological Co.,Ltd) for 2 h at room temperature to get crude extract (216 g). Crude extract was suspended in water (1500 mL), and then sequentially extracted with petroleum ether, dichloromethane (DCM), ethyl acetate and n-butyl alcohol (each 4500 mL). The ethyl acetate phase (8.4 g) was subjected to a silica gel column chromatography (100–200 mesh, 60 × 5.0 cm, flow rate 1.5 mL/min) eluted with a step gradient of CHCl_2_/MeOH (10:1, 8:1, 6:1, 5:1, 4:1, 2:1, 1:1 and 1:2, each 6 L) to yield 8 fractions(Fr.1 to Fr.8). Fr.7 (1.5 g) was applied to another cycle of the same silica gel column chromatography eluted with a step gradient of CHCl_2_/MeOH (10:1, 8:1, 5:1, 4:1, 2:1, and 1:1, each 2 L). Six fractions (Fr.7–1 to Fr.7–6) were collected. Fr.7–2 (230 mg) was further purified on a Waters autopurification system using a C18 reversed-phase column (XTerra Pre, 19*150 mm, 5 μm particle size, flow rate 3 mL/min) with a linear gradient of 0–90% methanol/water to yield 3 compounds (C1~C3). Purity of compound C2 (17 mg) was >99% as assessed by analytical HPLC (Waters alliance system) with C18 column (XTerra OBD, 2.1*150 mm, 5 μm particle size, USA).

### LC- MS analysis

LC- MS results were obtained on a Waters Alliance HPLC system consisting of a separations module (Waters 2695), a column oven and a PAD detector connected to a Waters Quattro Micro triple-quadrupole mass detector. LC-MS conditions were as follows:

Column: Xterra Ms C18 (5 μm, 2.1×150 mm; waters); solvent system: A—acetonitrile, B—0.2% acetic acid; gradient elution 10–40% A for 25 min, 40–90% A for 5 min, 90–100% A for 10 min; flow rate: 0.2 mL/min; injection volume: 10 μL; sample concentration: 0.5 mg/mL in acetonitrile; PAD conditions: spectra 190–700 nm; ESI-MS conditions: electrospray negative ion mode; scan range: 100–1000 amu; source temperature: 120°C; desolvation temperature: 250°C; capillary voltage 2.8 kV; cone voltage 50 V.

### CFTR-mediated iodide influx assay

The iodide influx fluorescence measurements in CFTR expressing FRT cells were done on a BMG plate reader (Fluostar Optima, BMG Lab Technologies) equipped with HQ500/20X (500 ± 10 nm) excitation and HQ535/30M (535 ± 15 nm) emission filters (Chroma Technology Corp., Brattleboro, VT) as references [10, 35]. Briefly, the FRT cells were seeded in 96-well black-walled-clear-bottomed tissue culture plates (Corning Inc., Corning, NY) at high density (20,000 per well) and incubated in CO_2_ incubator for 24–36 h until the cells were confluent. The cells were washed 3 times with PBS, prestimulated with a cocktail (5 μM FSK, 100 μM IBMX and 25 μM GEN in PBS) for 10 min, and then incubated with test samples for another 10 min. Iodide influx rate (d[I^-^]/dt) of each well was assayed by continuously recording fluorescence for 2 s (baseline) and 12 s after addition of 120 μL I^-^–containing PBS (in which 137 mM Cl^-^ was replaced by I^-^). The d [I^-^]/dt values were computed from fluorescence data by non-linear regression. The detailed assay and computation methods were described in the references [10, 41, 42].

### Rat colonic mucosal short-circuit current measurements

Short-circuit current (Isc) was measured as reference [10]. Briefly, Wistar rats (~200g) were sacrificed by one overdose intraperitoneal sodium pentobarbital (100 mg/kg). The colon was removed and washed with ice-cold Krebs buffer, and then stripped of muscularis and mounted in Ussing chambers (Physiological Instruments). The hemichambers were filled with symmetric solutions of a modified Krebs-bicarbonate solution containing (in mM): 120 NaCl, 5 KCl, 1 MgCl_2_, 1 CaCl_2_, 10 D-glucose, 5 HEPES, 25 NaHCO_3_, 0.01 indomethacin, and 0.01 amiloride, pH 7.4. The solutions were continuously bubbled with 95% O_2_/5% CO_2_ at 37°C.

### Intestinal fluid secretion measurements

*In vivo* efficacy of EGCG and ECG was evaluated in a closed-loop mice model of cholera toxin-induced intestinal fluid secretion. Briefly, KM mice (age 8–10 weeks) were deprived of food and free access to water for 24 hours. The mice were anaesthetized with intraperitoneal sodium pentobarbital (40 mg/kg). Three closed ileal loops (length 10–15 mm) proximal to the cecum were produced by sutures. Loops in each mouse were injected with saline alone, saline containing cholera toxin (0.5 μg) without or with 10 μg EGCG (or ECG). At 6 hours, the mice were sacrificed with one overdose intraperitoneal sodium pentobarbital (100 mg/kg), and intestinal fluid secretion was quantified from loop weight-to-length ratio. Details were described in reference [10].

### Statistical analysis

Data are expressed as the mean±SE or as representative traces. Student’s t test was used to compare test and control values, P values < 0.05 were considered to be statistically significant.
